# Piloting a Novel eHealth Technology for the Control and Management of Elevated Blood Pressure in Rwanda (HeartCare@Home Project): Protocol for a 2-Phase Crossover Study

**DOI:** 10.2196/66211

**Published:** 2025-12-17

**Authors:** Aurore Nishimwe, Juliette Gasana, Regine Mugeni, Celestin Twizere, Eric Hitimana, Odile Bahati, Olive Mukeshimana, Cedrick Manirafasha, Jean de Dieu Bugingo, Evariste Ntaganda, Francois Uwinkindi, Marc Twagirumukiza

**Affiliations:** 1 University of Rwanda Kigali Rwanda; 2 Kibagabaga District Hospital Kigali Rwanda; 3 Center of Excellence in Biomedical Engineering and eHealth, University of Rwanda, Kigali Rwanda; 4 College of Science and Technology, University of Rwanda Kigali Rwanda; 5 College of Medicine and Health Sciences, University of Rwanda Kigali Rwanda; 6 Eritcus Solutions Ltd Kigali Rwanda; 7 Rwanda Biomedical Center, Ministry of Health - Rwanda. Kigali Rwanda; 8 Faculty of Medicine and Health Sciences, Ghent University Ghent Belgium

**Keywords:** eHealth technology, eHealth innovation, heart care at home, hypertension, Rwanda

## Abstract

**Background:**

Effective blood pressure (BP) monitoring is vital for the management of hypertension, allowing timely adjustments in treatment. This study focuses on the development and implementation of an innovative, locally designed eHealth technology, the HeartCare@Home system, to enhance the control and management of hypertension in outpatient noncommunicable disease (NCD) clinics in Rwanda. The HeartCare@Home system comprises a mobile health app that incorporates rapid SMS technology, an integrated dashboard for signal reception at the clinic office level, and a clinical decision support algorithm.

**Objective:**

This study aims to assess the clinical efficacy, feasibility, and acceptability of a novel eHealth technology, the HeartCare@Home system, that uses home and clinic-based automated BP monitoring with real-time management of elevated BP in an outpatient NCD clinic in Rwanda.

**Methods:**

This pilot study will use an interventional design with a crossover approach to test the clinical efficacy of the HeartCare@Home system at the NCD clinic of Kibagabaga District Hospital. A total of 140 patients with hypertension will take part in the study. All enrolled patients will be allocated to either the interventional group or the standard of care group. The follow-up for each group will be 6 months (3 months in each group follow-up). The data for the intervention group will be generated by our mobile health app, while data for the control group (standard care) will be retrieved from usual patient files at the NCD clinic. Data extraction sheets will be used for standard care data retrieval. The Feasibility of Intervention Measure and Acceptability of Intervention Measure tools will be used to cross-sectionally evaluate the feasibility and acceptability of the HeartCare@Home system. Data will be summarized with descriptive statistics. A paired sample 2-tailed *t* test will be used to test for differences between the pre- and postintervention records for hypertension control.

**Results:**

This study will yield a technical infrastructure, the HeartCare@Home system, to support the control and management of hypertension in outpatient NCD clinics. It will introduce a new model of health care delivery through innovative technology that enables home-based BP monitoring. This will offer a unique technology to enable elevated BP control and timely hypertension management and will also ensure a real-time communication linkage between patients and the appropriate level of care. Furthermore, the findings from the assessment of the clinical efficacy, feasibility, and acceptability of the HeartCare@Home system will inform possible scalability of the system to more NCD clinics. The study is currently in the implementation stage.

**Conclusions:**

The HeartCare@Home project will address the important gap of low BP control rates in patients with hypertension, which has contributed to delayed consultations and increased cardiovascular mortality. Such eHealth technology infrastructure may also be scalable to other settings.

**International Registered Report Identifier (IRRID):**

DERR1-10.2196/66211

## Introduction

### Background

Cardiovascular disease (CVD) is the leading cause of mortality globally, accounting for 17.9 million deaths each year, and hypertension is the leading risk factor associated with CVD [1,2]. In low- and middle-income countries of sub-Saharan Africa with rapid epidemiological, demographic, sociocultural, and economic transitions, the risk of hypertension results in more than 80% of deaths annually [3]. In Rwanda, the prevalence of hypertension is approximately 15.3%, with rates as high as 40% in individuals aged 55 to 64 years [4]. According to the 2019 National Institute of Statistics in Rwanda projections, an estimated 1.1 million Rwandan people aged between 15 and 69 years have hypertension [4]. However, data from the national health management information system show that only 70,200 individuals, approximately 6.3% of patients with hypertension, receive care at noncommunicable disease (NCD) clinics. Therefore, more effective and rapid hypertension control interventions are essential to reduce CVD-related morbidity and mortality.

Effective hypertension control intervention measures, such as blood pressure (BP) monitoring for early clinical decisions to seek care and access to evidence-based interventions for early management of hypertension, are still limited in Rwanda [4]. Geographical barriers such as long distances to health services, prolonged waiting times, and inadequate patient follow-up may lead to late diagnosis of hypertension, poorly managed hypertension, and increased risk for CVD. In clinical practice, physicians often check hypertension status and make medication decisions based on 1 or 2 instant measurements despite the known variability of BP [5]. There is a need for more focused and personalized decision-making supported by richer data, such as daily BP measurements.

**Figure 1:**
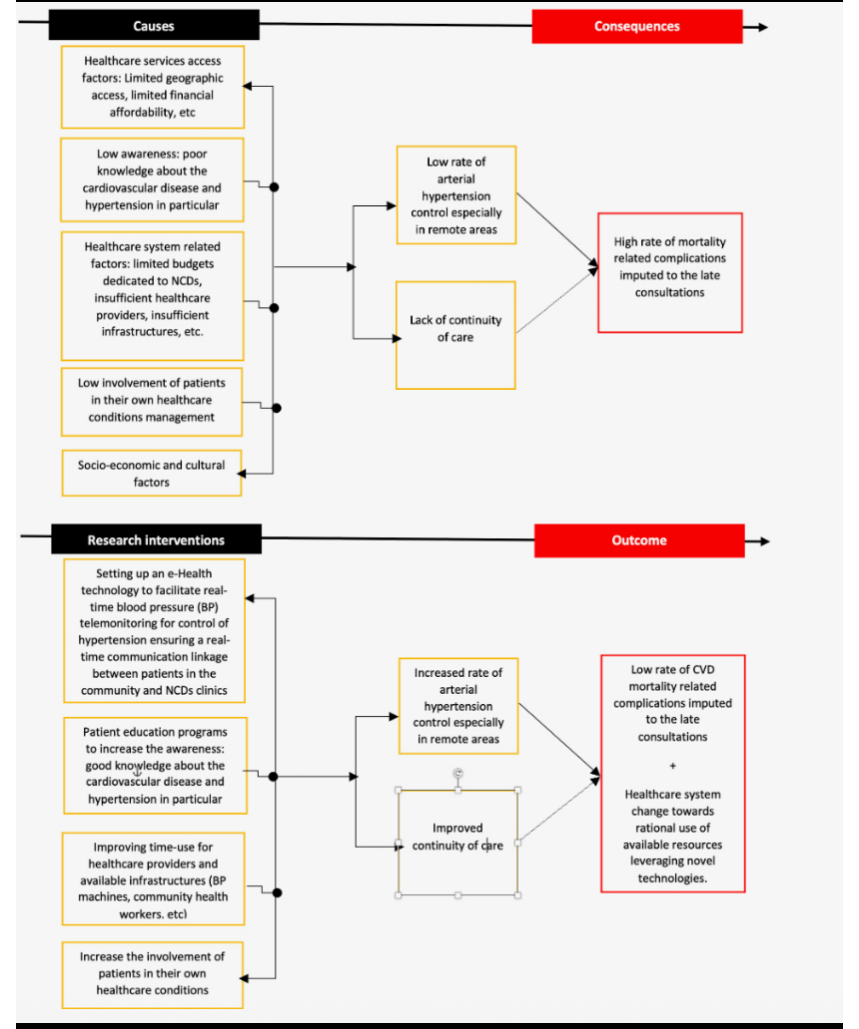
Theory of change.

Increasing evidence supports the use of eHealth solutions to control hypertension by collecting high-volume data from rural and urban community-based patients for quick clinical decision-making [6,7]. eHealth tools support improved BP monitoring and contribute to increased adherence to antihypertensive treatments, thus improving early effective BP control [8,9]. However, the role of team-based care for BP control involving task-shifting benefits and resources that constitute the complex procedure carried out in a BP telemonitoring are not well documented [10-13]. There is evidence for improved health by hypertension control through BP telemonitoring [14]. Furthermore, the role of an eHealth intervention model in achieving adequate hypertension control requires further exploration within the Rwandan context, given the country’s diverse social and cultural beliefs. Research on the implementation outcomes of eHealth technology for BP telemonitoring could inform interventions for improved policy and practice in Rwanda. Therefore, this study aims to pilot test and investigate the clinical efficacy and feasibility of a novel eHealth technology, the HeartCare@Home system, with real-time BP telemonitoring for control of hypertension in Rwanda. What distinguishes this solution from existing market offerings is its fully homegrown design, making it both affordable and accessible for Rwandans. Its local origin also ensures easier local maintenance and local support, promoting readiness for integration into the Rwandan health system. Furthermore, the system’s dashboard and the clinical decision support (CDS) algorithm are tailored specifically to the Rwandan context, enabling real-time data reception and patient monitoring. Figure 1 describe the proposed theory of change of this project.

### Aims

The specific aims of the project are as follows:

1. To assess the clinical efficacy of an innovative eHealth technology (home and medical automated evaluation of the BP with live management of elevated BP) in an outpatient NCD clinic in Rwanda. For this aim, we will be hypothesizing that patients using HeartCare@Home system will have a lower systolic and diastolic BP and higher control rates than individuals in standard of care.
2. To determine the feasibility and acceptability of the eHealth technology in an outpatient NCD clinic in Rwanda.

### Description of the HeartCare@Home System, a Home-Based BP Telemonitoring Novel Technology in Rwanda

The HeartCare@Home system is an eHealth technology that enables remote data transmission of BP measures and additional information on patients’ health status from their homes to the physician’s office or the hospital. This project will use 4 main novel technologies to facilitate real-time BP telemonitoring for control of hypertension, ensuring a real-time communication linkage between patients in the community and the appropriate level of care (NCD clinics). These main technologies include a rapid SMS gateway technology embedded in a mobile health (mHealth) app, a central database, and an integrated dashboard with the existing medical record system (OpenMRS), a CDS algorithm that will send BP measurements with signal outputs (color codes) with red flags on abnormal BPs measurements, and internet of things technology to support communication between the central database and the mHealth app.

The HeartCare@Home system will involve individual patients and community health workers (CHWs) taking BP measurements at the community level and sending the measurements to an allocated physician using an mHealth app. The patients and CHWs will use the mHealth app to record BP measurements twice weekly and send them to the central server located at the hospital. The mHealth app will also be complemented by the provision of BP machines for daily BP measurements. The mHealth app will also be used as a reference tool to upgrade patients’ health literacy regarding hypertension self-management.

**Figure 2:**
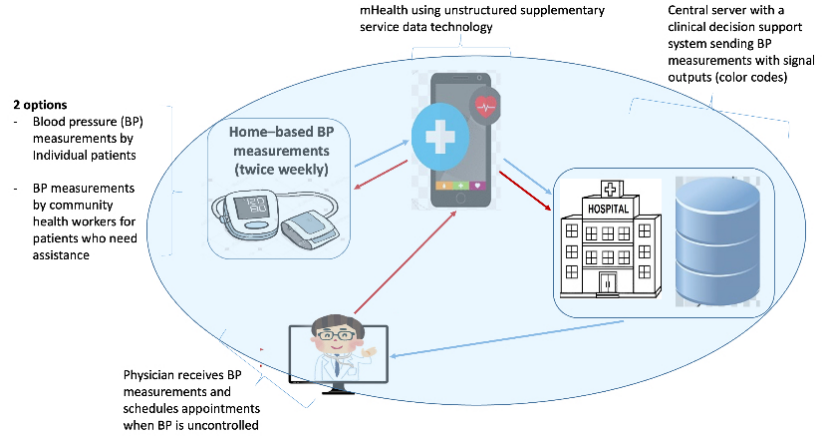
HeartCare@Home system prototype. BP: blood pressure; CDDS: clinical decision support system; CHW: community health worker; mHealth: mobile health.

In addition, the central server will display the recorded BPs on an integrated dashboard (integrated within OpenMRS and linked to patient health records) using a CDS algorithm that displays alerts (red flags) for the abnormal BPs in the OpenMRS used by NCD clinics to enable prompt clinician notification and timely action based on real-time BP measurements. The clinicians will be able to initiate physical appointments for patients with uncontrolled BPs based on real-time BP measurements. The caring physicians will also be able to initiate and adjust hypertension drug treatment based on real-time BP measurements. Figure 2 shows the HeartCare@Home prototype.

## Methods

### Study Setting

The study will be conducted in the NCD clinic of Kibagabaga District Hospital located in Gasabo District, Kigali, Rwanda. This study site was selected as a potential piloting setting because of the high number of patients with hypertension enrolled in the NCD clinic.

### Study Population

This research will enroll individual patients with known diagnosis of hypertension, aged 45 to 65 years, from the Gasabo District community enrolled in the Kibagabaga Hospital’s NCD clinic. All study participants who will be willing to participate in the study will be continuously linked to the NCD clinic for further processing of BP measurement data. Two internist physicians working in the NCD clinic will also be involved in the study. The NCD clinic was selected for logistical feasibility, as it has been one of the first clinics in Rwanda to provide hypertension care services for more than 3 years.

### Sample Size Calculation

The study will enroll 140 participants diagnosed with hypertension, aged between 45 and ≥65 years, from the community of Gasabo District in Kigali, Rwanda. The sample size was estimated based on testing hypothesis 1, using the approach for sample size calculation for comparison between 2 groups when the end point is quantitative data [15]. The expected difference in BP (both systolic and diastolic) will be fixed at 3 mm Hg (effect size of 0.20). The level of significance was fixed at 5%, and the power of study was fixed at 80%.

### Study Design

**Figure 3:**
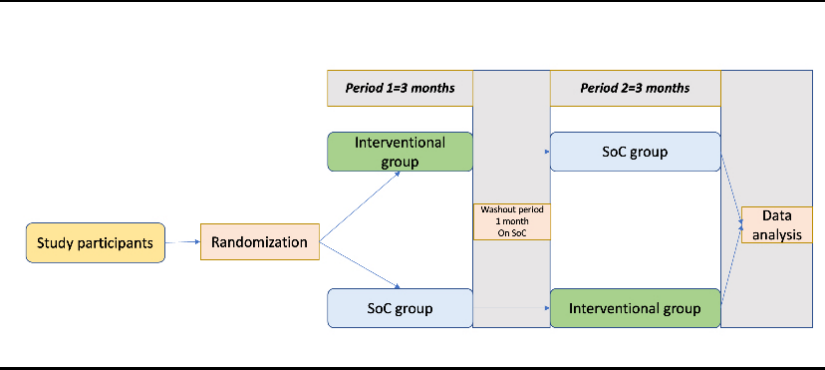
Study design (hypothesis 1). SoC: standard of care.

**Figure 4:**
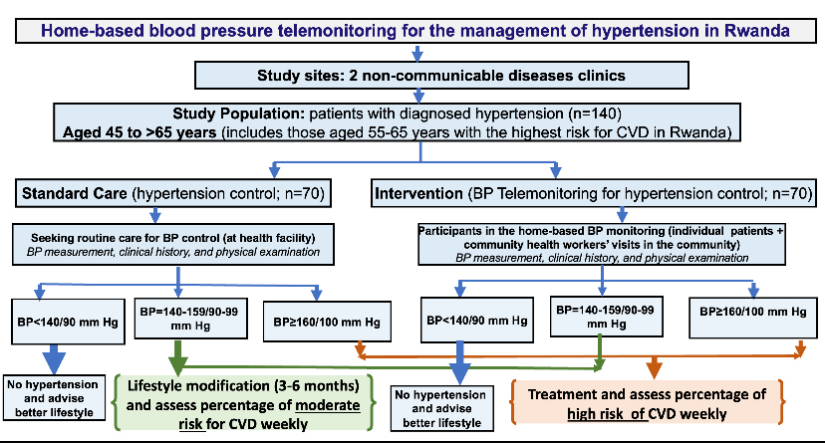
Flowchart for the comparison of the standard care and the home-based blood pressure (BP) telemonitoring program. CHW: community health worker; CVD: cardiovascular disease.

This study will use an interventional design with a crossover approach to test the clinical efficacy hypothesis. All identified patients will be allocated to either the intervention group or the standard of care group. The follow-up for each group will be 6 months (3 months in each group follow-up). A comparison of the standard care and the home-based BP telemonitoring program will be conducted using a record review approach. The data for the intervention group will be generated by our mHealth app, while data for the control group (standard care) will be retrieved from usual patient files at the NCD clinic. Data extraction sheets will be used for standard care data retrieval. Figure 3 illustrates the study design for testing the clinical efficacy of the HeartCare@Home system, while Figure 4 shows the flowchart for the comparison of standard care and the home-based BP telemonitoring program. The specific outcomes will be evaluated using variables such as BP values before and after the intervention, which will guide clinical recommendations, including lifestyle modifications, cardiovascular risk assessment, and treatment.

To assess the feasibility and acceptability of the HeartCare@Home system, a cross-sectional study methodology using Likert-scale questionnaires, the Feasibility of Intervention Measure and Acceptability of Intervention Measure [16], will be adopted. The outcome measures will include measures of acceptability and feasibility, such as perceived approval, likability, and willingness to adopt the system.

### Recruitment of Study Participants

Study participants will be identified by the researcher in collaboration with district and village administrators and CHWs in consideration of their hypertension diagnosis and age. Both genders will be included. Participants will be invited through verbal communication and written letters.

### Data Collection

We will conduct a preintervention record review of hypertension control data. The intervention entails the implementation of a home-based BP telemonitoring program for a period of 6 months in the Gasabo District communities. A total of 6 months after the intervention, the clinical efficacy of the home-based BP telemonitoring program will be evaluated by comparing the rate of hypertension control during the BP telemonitoring program implementation with the previous rate of hypertension control within usual care using a record review approach. The standard BP measurement at home procedures will be followed. These include having the patient sit in a comfortable chair with their back supported for at least 5 minutes before the reading, placing both feet flat on the ground with legs uncrossed, resting the arm with the cuff on a table at chest height, and then measuring the BP [5]. On the basis of the implementation research framework by Proctor et al [17], we will evaluate the feasibility and acceptability of the home-based BP telemonitoring program. The Acceptability of Intervention Measure and the Feasibility of Intervention Measure [16] tools will be used for data collection. The original tools will be translated into Kinyarwanda. Both the original and the translated versions of the tools will be used to collect data. Informed consent will be obtained from each study participant before the start of data collection. CHWs will participate as data collectors and remunerated for their time and effort as per the Ministry of Health guidelines.

### Data Management and Analysis

Data analysis will be conducted using Stata (StataCorp LLC) software. Double data entry will be used to maximize the accuracy of data entry. Data cleaning will be carried out using the study questionnaire to check whether data from participant’s answers match with the answers that will be in the questionnaire. Data will be summarized with descriptive statistics. Cleaned data would be cross-tabulated to determine relationships between different variables. A paired sample 2-tailed *t* test will be used to test for differences between the preintervention and postintervention records review data for hypertension control.

### Ethical Considerations

Ethics approval has been obtained from the Rwanda National Research Ethics Committee (06/RNEC/2024). Approval has also been sought from the hospital authorities to access electronic medical records. Informed consent will be sought from all the study participants before participation in this study. Anonymity will be ensured by not collecting participants’ names and instead using pseudonyms. This study will be carried out in accordance with relevant guidelines and regulations outlined in the ethical declarations.

## Results

### Expected Study Results

This pilot project aims to induce a long-term change in the health care delivery system in Rwanda and in other similar settings with a new way of monitoring and managing patients with NCD. In addition, the results from evaluating the clinical efficacy, feasibility, and acceptability of the HeartCare@Home system will guide its potential expansion to additional NCD clinics. The overall ambition is to complete a successful 2-year pilot phase and subsequently expand the system nationwide in Rwanda, with the potential to include other NCDs.

### Innovation and Impact

This project supports scientific and technological advancement as Rwanda endeavors to become a middle-income country by 2050. The project will use 4 main novel technologies to facilitate real-time BP telemonitoring for control of hypertension, ensuring a real-time communication linkage between patients in the community and the appropriate level of care (NCD clinics), as demonstrated in Figure 2.

### Community Outreach Plan

This research will facilitate a real-time communication linkage between patients at the community level and the appropriate level of care. Community outreach is essential for this study to effectively engage the Rwandan community and raise awareness about the project. A detailed outreach plan will be prepared in the work package 5 to create awareness of the project’s rationale within the Rwandan society and later disseminate study findings. Information about this study will be communicated to citizens through Rwanda’s health system communication channels and CHWs.

### Dissemination of Study Results

The dissemination and exploitation activities plan will mainly focus on the elevated BP handling gap in Rwanda and the need for Rwandan homegrown solutions. This has already been initiated with a project kickoff meeting, CHWs meetings, and series of workshops and technical development hackathons. The aim of the dissemination will be to ensure the optimal use of the project’s findings after its completion and therefore speed up the potential for their uptake by health care providers, researchers, regulators, and policymakers. This research project consortium includes the Rwanda Biomedical Centre, a technical body of Ministry of Health in Rwanda, which will ensure that the project outcomes contribute to the Government of Rwanda priorities and needs in terms of NCD prevention and management. In addition, this project through the work package 5 will use a multilevel approach, including tailor-made dissemination tools and activities depending on the respective target audiences and their needs. The dissemination plan also includes the organization and coordination of all means for project dissemination and outreach, such as scientific articles, oral or posters presentations, meetings, seminars, and scientific conferences. We anticipate publishing findings of this study in peer-reviewed journals. A copy of the published paper or abstract outlining the study findings will be shared at local and international academic forums and meetings. The study findings will also be presented to hospital administrators and community members.

## Discussion

### Anticipated Findings

The study will primarily develop a novel Rwandan, homegrown eHealth technology as a ready-to-use, software-as-a-service, marketable product comprising different packages (an mHealth app with rapid SMS gateway, a central server and an integrated dashboard, and a CDS algorithm) that will facilitate health care services for the control of hypertension among individuals diagnosed with hypertension in communities of Rwanda. The main outcome from the HeartCare@Home system innovation will be to enable a change in health care delivery for patients with hypertension in Rwanda, improving monitoring, early detection of BP worsening, reducing hypertension complications and cardiovascular risk, and allowing health care providers to handle more patients in less time.

After pilot testing and establishing the eHealth technology system as operational in 1 NCD clinic, further large-scale research will be needed to evaluate its scalability across Rwanda and potentially in other similar settings in sub-Saharan Africa. This is a crucial contribution to health care systems and science, particularly in the African region, which is currently facing emerging NCDs, including hypertension. Additionally, the lessons learned from the HeartCare@Home project may inform the establishment of a scalable system that can be applied in other disease areas. The HeartCare@Home system may also be adaptable to other cardiovascular conditions, such as arrhythmias, or NCDs, integrating additional home or portable electrocardiograms devices, and for NCDs (integrating other wearable devices).

### Anticipated Research Limitations

This study will not generalize findings, and it will recruit a relatively small sample (n=140 participants). However, the findings from this study will inform a larger study aimed at evaluating the implementation outcomes of the home-based BP telemonitoring program on a large scale.

## Data Availability

The dataset generated for some of the phases of the study has not yet been analyzed but will be made available from the corresponding author upon reasonable request with the necessary permissions in line with data protection regulations.

## Appendix

Multimedia Appendix 1 Peer review report from the Vlaamse Interuniversitaire Raad, Universitaire Ontwikkelingssamenwerking (VLIR-UOS) / Flemish Interuniversities Council, University Development Co-operation (Belgium).

## Abbreviations

BP: blood pressure
CHW: community health worker
CVD: cardiovascular disease
mHealth: mobile health
NCD: noncommunicable disease
