# Supplementary material for: Piloting a Novel eHealth Technology for the Control and Management of Elevated Blood Pressure in Rwanda (HeartCare@Home Project): Protocol for a 2-Phase Crossover Study
Source: JMIR Res Protoc. 2025 Dec 17;14:e66211. doi: 10.2196/66211 (PMC12756656; doi:10.2196/66211)
Supplement: Multimedia Appendix 1 [file resprot_v14i1e66211_app1.pdf]

Selection Commission Africa 1

Short Initiatives (SI) 2022

Extract

Rwanda

VLIR-UOS project number	Type	Flemish promoter	Local promoter	Flemish institution	Partner institution	Project Title
341-SI 2022	SI	Marc Twagirumukiza	Aurore Nishimwe	Universiteit Gent	University of Rwanda	Piloting a novel, scalable, eHealth technology for the control and management of elevated Blood Pressure in Rwanda (HeartCare@Home Project)

+	-
• A very relevant and well-designed project, addressing the human resources gap by eHealth technology. It shows an excellent dissemination strategy for good local ownership and corresponds to well-identified needs.<br>• The project presents a realistic and applicable ToC with expected positive impact on the healthcare system and with expected benefits for patients. Based on the outcome of the project further introduction of eHealth technology can be planned.<br>• The implementation planning is detailed and seems feasible (but optimistic.) The mix of partners is solid and they know each other from previous projects. Also partners from the business world are involved.<br>• The new technology is focused on vulnerable audiences with a very good dissemination approach.	• A clearer presentation of the mode of governance would make it possible to better understand the active participation of all stakeholders, including the Ministry of Health (main political actor to introduce the application in Rwanda).<br>• The proposal has real short-term impacts for diagnosing patients with elevated blood pressure. This is a pilot project, it is difficult to measure the long-term impacts that depend on the implementation of this technological system.<br>• The consideration for gender and LNOB (vulnerable persons) is not visible and needs to be developed.<br>• An old budget format has been used. An updated one needs to be filled out and submitted.

Conclusions (incl. flags/main attention points): **Project is selected** - This is a well presented project but the consideration for gender and LNOB (vulnerable persons) is not visible and needs to be developed.. Also the management plan of the interactions of the different stakeholders and the (essential) role of the Ministry of Health should be more explicit.The budget format needs to be updated.
